# Solar photocatalysts: non-metal (C, N, and S)-doped ZnO synthesized through an industrially sustainable *in situ* approach for environmental remediation applications†

**DOI:** 10.1039/d4ra03492a

**Published:** 2024-07-08

**Authors:** Amala Joy, Mangalaraja R. Viswanathan, Baiju K. Vijayan, Claudia G. Silva, Irfana Basheer, Sreejamol Sugathan, Peer A. Mohamed, Ananthakumar Solaiappan, Anas Shereef

**Affiliations:** a Department of Chemistry, T. K. M. College of Arts and Science, Research Centre, University of Kerala Kerala India anas@tkmcas.ac.in; b Facultad de Ingeniería y Ciencias, Universidad Adolfo Ibáñez Diag. Las Torres 2640, Peñalolén, Región Metropolitana 7941169 Santiago Chile; c Department of Chemistry/Nanoscience, Kannur University Swami Anandha Theertha Campus Payyannur Kerala India; d Laboratory of Separation and Reaction Engineering – Laboratory of Catalysis and Materials (LSRE-LCM), Faculdade de Engenharia, Universidade do Porto Rua Dr Roberto Frias S/n 4200-465 Porto Portugal; e Materials Science and Technology Division (MSTD), National Institute for Interdisciplinary Science and Technology (NIIST), Council of Scientific and Industrial Research (CSIR) Trivandrum Kerala India

## Abstract

One of the biggest issues the world is currently experiencing is the scarcity of pure water due to the contamination of pure water by human activities. Highly efficient, semiconducting photocatalytic materials have great potential as future catalytic materials for facilitating the clean-up process of contaminated water. Among the many semiconductor photocatalysts, non-metal-doped zinc oxide (ZnO) nanoparticles have attracted special attention in the scientific field for environmental remediation applications. The present paper reports an easy and viable synthesis of C-, N-, and S-based ZnO semiconductor photocatalysts through a simple heating method. The structural changes in the obtained samples were studied using XRD, TG/DTA, and FT-IR analyses, and morphological examinations were performed using TEM and SEM. The quantification of non-metal dopants was carried out using CNS and XPS analyses. The surface areas of the samples were analyzed using the BET method and the band energies of the samples were measured using UV-vis-diffuse reflectance Kubelka–Munk plots. Photoactivity studies were performed and revealed that the utilized *in situ* method resulted in the development of high-performance sulphur – (81.4%, *k* = 1.951 × 10^−2^ min^−1^), nitrogen – (78.5%, *k* = 2.271 × 10^−2^ min^−1^), and carbon – (67.2%, *k* = 1.392 × 10^−2^ min^−1^) doped ZnO photocatalysts. As revealed through XPS and UV analyses, a possible electron-transfer mechanism is suggested, wherein electronic transition occurred from different sub-bands when non-metal elements were introduced into the ZnO lattice. The study paves the way for the bulk-scale fabrication of doped nanoparticles through a simple heating method, whereby the unique combination of the present method with bandgap engineering will ultimately produce advanced non-metal-based ZnO photocatalysts that could find useful applications in sustainable industrial sectors.

## Abstract 2

Nanocrystalline, non-metal (C, N, and S)-doped ZnO spherical clusters are successfully synthesized through a sustainable thermo-evolution method. Solar photocatalysts with high catalytic efficiency in the order C-ZnO < N-ZnO < S-ZnO are prepared.

## Graphical abstract

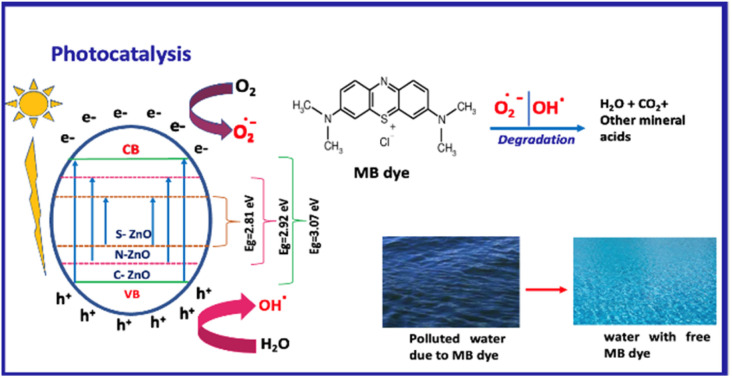

## 1 Introduction

ZnO and its derivatives have emerged as a central research topic in scientific investigations in recent years, and these materials have been highlighted for their potential application in different fields such as medicine, cosmetics, textiles, food, electronics, materials sciences, optics, ceramics, and drug delivery.^1–5^ The wide application of these materials could be realized through their unique properties, such as wideband gap,^6^ oxygen-deficient nature,^7^ splendid morphological availability,^8^ large surface area,^9^ strong adsorption ability,^10^ less toxic nature,^11^ good biocompatibility,^12,13^ and high anti-microbial activity.^14^ Among these, the photocatalytic property of ZnO has shown promising potential for many environmental applications.^15–18^ Since semiconducting ZnO has a large bandgap (>3.0 eV), it gets excited in the wavelength range of 100–400 nm, *i.e.* in the UV region of the electromagnetic spectrum.^19–21^ By further modifying ZnO, such as doping with metals or non-metals, it can be converted from a UV-active material to an efficient solar photocatalyst that can absorb UV and visible light.^22–25^ In addition, by including dopants, these materials can be used as solar photocatalysts for environmental remediation applications and the treatment of wastewater and coloured effluents/dyes in industrial water.^26–29^

Doping ZnO with non-metals (N, C, S, F, *etc.*) can increase the semiconductor photocatalytic ability of ZnO by improving the carrier's mobility rate and the efficiency of charge separation.^30–36^ For example, work carried out in a similar line showed that carbon-doped ZnO nanostructures displayed superior visible-light photocatalytic degradation towards methylene blue (MB) dye due to the increase in the separation efficiency of photogenerated charge carriers.^30^ Moreover, nitrogen (30%)-doped ZnO was reported to be an efficient photocatalyst for removing organic herbicides (2,4-D and picloram) under visible-light irradiation, due to its ability to decrease the combination of electron–hole pairs.^31^ The doped ZnO nanostructure was shown to play an essential role in the degradation of phenol and 2,4-dichlorophenol under visible light than that of the bare ZnO.^32^ The N-doped ZnO catalyst prepared by the hydrothermal method was reported to act as an efficient photocatalyst for the degradation of methylene blue from wastewater samples, and the degradation products of MB dye were also assessed.^33^ In another study, ZnO doped with nitrogen supported on graphene oxide showed high photocatalytic performance for MB degradation, acting as a hindrance to recombination and promoting electrons towards the conduction band. In addition, the catalyst also acted as a collector and an electron transporter.^34^ The coupling of ZnO with S-doped graphitic carbon nitride (g-C_3_N_4_) was also reported to possess enhanced photocatalytic activity.^35,36^

Hence, by taking account of all the positive aspects of doping non-metals to ZnO, herein we adopted an industrially viable thermo-evolution method to prepare doped ZnO nanoparticles, with the use of non-metal dopants, such as carbon, nitrogen, and sulphur. Our prior report of this work described the unique gelation of zinc acetate in tartaric acid systems.^37^ In a short communication paper, we reported an accelerated microwave method for preparing non-metal doped ZnO.^38^ The present paper provides detailed information about a facile *in situ* thermo-evolution synthesis method for the morphologically controlled synthesis of non-metal-doped ZnO nanoparticles. Specifically, an industrially viable and sustainable approach is detailed here for the synthesis of carbon-, nitrogen-, and sulfur-doped ZnO. The unique merits of the synthesis method, in terms of the sample's crystallite size, structural modifications, chemical composition, surface area, and pore-size distribution, are addressed in detail. The step-wise morphological evolution of spherical nanoclusters of doped ZnO from a thermally unstable nanowire precursor gel is also addressed. Full evidence of successfully obtaining the doped nanoparticles was provided through different characterization studies, including XRD, TG/DTA, SEM, TEM, UV–visible, and BET analyses. The structurally bound dopants on ZnO were confirmed through FT-IR, XPS, and CNS analyses. For environmental remediation applications, the sunlight-active photocatalytic ability of the prepared samples was analyzed by evaluating the degradation of methylene blue dye under UV–visible light illumination from 0 to 180 min. Band gap engineering studies of the samples were performed, and the results were correlated to explain the mechanism behind the high-performance photocatalytic activity. A comparative photocatalytic performance analysis was also carried out among the synthesized samples, and the mechanism for the high-performance photocatalytic activity was proposed and explained in detail.

## 2 Experimental

### 2.1 Chemicals and reagents

Zinc acetate dihydrate [98.9%, (CH_3_COO)_2_Zn·2H_2_O] and methylene blue [98%, (C_16_H_18_C_1_N_3_S)] were purchased from Merck, India. Tartaric acid [>99.7%, (C_4_H_6_O_6_)], ethanol [>99.9%, (CH_3_OH)], urea [99%, (NH_2_CONH_2_)], and thiourea [98.9%, (NH_2_CSNH_2_)] were purchased from Sigma-Aldrich, USA. These reagents were used without any further purification. All the solutions were prepared with double-distilled water.

### 2.2 Synthesis of C-doped zinc oxide nanoparticles

First, 2.25 g of tartaric acid was precisely weighed into a 50 ml beaker, and 30 ml (0.6 M) of ethanol was introduced into it. The solution was then stirred for 10 min using a magnetic stirrer. Next, 3.2923 g of zinc acetate dihydrate was dissolved in 30 ml (0.6 M) of ethanol in a 100 ml beaker, which was then stirred for 30 min. The tartaric acid solution was added dropwise into the solution of zinc acetate to gradually form a white-coloured opaque gel, which was allowed to stabilize and then dried in an air oven at 100 °C for 3 h. Finally, the obtained product was crushed in an agate mortar and thermally treated separately at 200 °C, 300 °C, 400 °C, and 500 °C for 3 h.

### 2.3 Synthesis of N-doped zinc oxide nanoparticles

Briefly, 2.25 g of tartaric acid was added to 30 ml (0.6 M) ethanol in a 50 ml beaker and the solution was magnetically stirred for 5 min. About 1.9754 g of zinc acetate, along with 15 ml (0.6 M) ethanol, were placed in another 100 ml beaker, and the solution was stirred for 30 min using a magnetic stirrer. Typically, to the zinc acetate solution, 0.6 M (0.54 g) urea in 15 ml ethanol and the tartaric acid solution were added to obtain a stable gel. The resulting gels were further aged and dried at 100 °C for 3 h and crushed in an agate mortar. The samples were then thermally treated at 200 °C, 300 °C, 400 °C, and 500 °C for 3 h.

### 2.4 Synthesis of S-doped zinc oxide nanoparticles

First, 2.25 g of tartaric acid was precisely weighed and dissolved in 30 ml (0.6 M) ethanol in a 50 ml beaker, and the solution was stirred for 10 min using a magnetic stirrer. Meanwhile, 1.975 g of zinc nitrate was added to 15 ml (0.6 M) ethanol in a 100 ml beaker and magnetically stirred for 30 min. To this solution, 0.6 M (0.685 g) of thiourea dissolved in 20 ml ethanol and the tartaric acid solution were added dropwise under stirring. The resulting gels were further aged and dried at 100 °C for 3 h. After that, the product was crushed in an agate mortar, and finally thermally treated at 200 °C, 300 °C, 400 °C, and 500 °C for 3 h.

### 2.5 Photocatalytic tests

The photocatalytic degradation of MB was conducted with the aid of UV–visible light illumination in a photoreactor using non-metal doped ZnO as a photocatalyst. The experiment used an AC of 220 V, supplied by 50 W UV and visible lamps. The dye and photocatalyst solution mixture was kept above a magnetic stirrer under the lamps at a distance of 10 cm. In this process, the synthesized photocatalyst (0.05 g) was added to MB dye solution (100 ml) in a beaker, and the solution was stirred well in the absence of light for 30 min to attain adsorption–desorption equilibrium. After that, with light exposure and at regular intervals, 5 ml of dye solution was taken out and analyzed by UV–visible spectroscopy to investigate the concentration of MB in the solution after the doped ZnO catalyst was separated using a PVDF filter. As time progressed, the colour of the MB faded with the photocatalyst, and the experiment continued until the colour of MB fully disappeared. The degradation profile of MB was obtained using a UV-Vis spectrophotometer.

### 2.6 Characterization techniques

The formed ZnO nanoparticle's crystalline nature was studied using X-ray diffraction (XRD). The XRD analyses of the corresponding samples were carried out with an XRD Rigaku mini flex system (Japan), as *λ* = 0.15406 nm, and operated at a voltage of 30 kV and current of 10 MA in the 2*θ* range from 20°–80°. The prepared ZnO nanoparticles' optical properties were characterized by UV-Vis absorption spectroscopy (Shimadzu UV-Vis spectrophotometer, Japan). The presence of functional groups and the structural features were studied using a Shimadzu IR Affinity FT-IR spectrophotometer (Japan), in the range 4000–400 cm^−1^. The size of the ZnO nanoparticles was measured by transmission electron microscopy (TEM JEOL, 200CX) at an operating voltage of 50–300 keV. The surface morphology and elemental analysis of the pure and doped ZnO were studied by scanning electron microscopy (SEM, JEOL, JSM-6700). The pore-size distribution and surface area of the samples were determined by a BET surface area instrument (Micromeritics Gemini 2370). Thermal analysis of the gel was carried out using TG/DTA/DTG instruments (Shimadzu, Japan) operated up to 1000 °C. The presence of dopants in ZnO nanoparticles was assessed using a CNS analyzer (PerkinElmer, Series-2-2400). The surface composition and proper doping of elements in the prepared samples were determined by X-ray photoelectron spectroscopy (XPS, PHI 5000 Versa Probe II USA).

## 3 Results and discussion

### 3.1 Structural and thermal studies

**Fig. 1:**
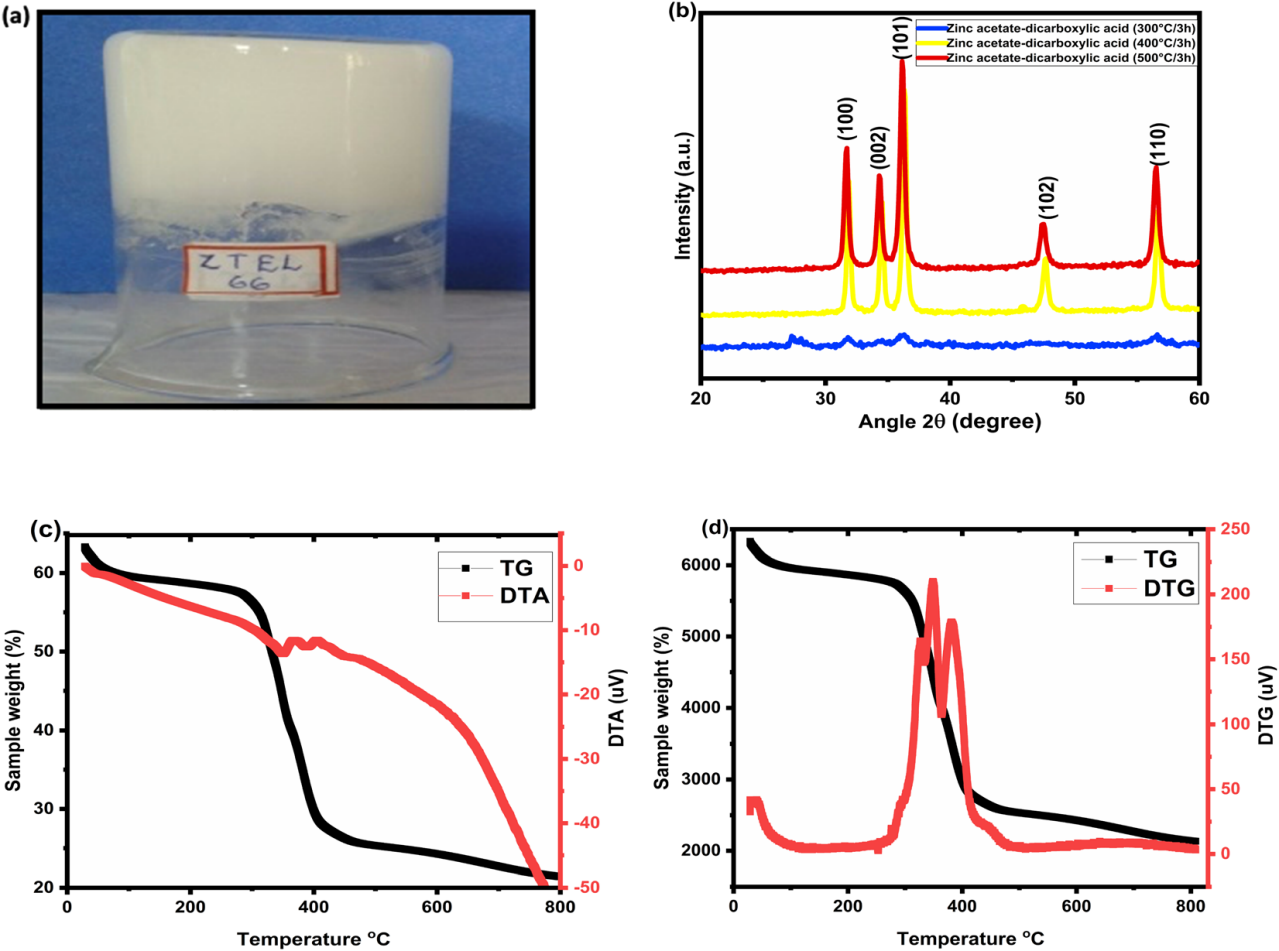
(a) Photograph of the stable gel formed from 0.6 M zinc acetate and 0.6 M tartaric acid in ethanol medium. (b) X-ray diffraction analyses of the thermally derived zinc oxide samples at different temperatures. (c) Thermal analyses of the gels and TG/DTA plot. (d) TG/DTG plot of the gels.

Fig. 1a shows the stable and hard gel formed from a 1 : 1 molar concentration of zinc acetate and tartaric acid (0.6 M each) in an ethanol medium. The structural features of the obtained gels were studied by XRD, TG, and DTA. Fig. 1b shows the X-ray diffraction patterns of the prepared gel samples treated at different temperatures of 300 °C, 400 °C, and 500 °C, respectively. There were no well-defined peaks corresponding to ZnO that could be detected at 300 °C, but in the case of the 400 °C- and 500 °C-treated samples, several diffraction peaks were noticed from the different atomic planes of ZnO. The crystalline nature of ZnO nanoparticles was evident from the X-ray diffractograms at 400 °C and 500 °C. The diffractograms were well indexed with the matching peaks of JCPDS file no. – 79-0205 for hexagonal wurtzite ZnO.^39^ The thermal analyses of the gels (TG/DTG/DTA) are depicted in Fig. 1c and d. The thermogravimetric (TG) analysis exhibited a three-step weight loss with three different decompositions, as seen in the graph. The weight loss due to the elimination of free ethanol and water molecules was evident from the graph by the first weight loss section of 6% at up to 290 °C. The huge weight loss of 50% from 290 °C to 400 °C was due to the decomposition of the gel network and ZnO formation, whereas the two differential peaks at 360 °C and 390 °C in the DTG analysis were due to the decomposition of the gel network and the formation of ZnO, respectively. From the thermal analysis and X-ray analyses, it was evident that a conventional heating of 400 °C was only required to form the ZnO nanoparticles.

### 3.2 Morphological studies

**Fig. 2:**
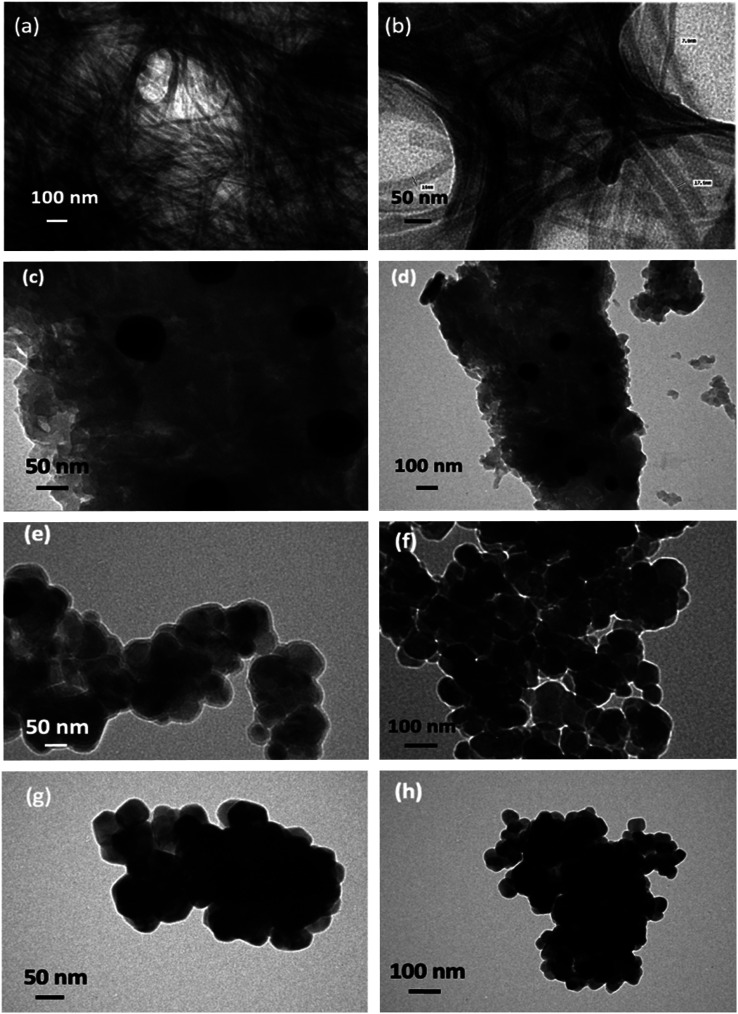
TEM images of the zinc acetate–tartaric acid (1 : 1) gels (a) and those prepared at (b) 200 °C/3 h, (c) and (d) 300 °C/3 h, (e) and (f) 400 °C/3 h, (g) and (h) 500 °C/3 h at different magnifications.

The bulk and fine morphologies of the prepared samples were studied by TEM and SEM analyses, respectively. TEM studies (Fig. 2) were carried out for the thermally treated gel samples from 200–500 °C to study the structural transformation happening in the gel. The morphological examinations were performed for the gel derived from a 1 : 1 molar concentration of zinc acetate and tartaric acid in ethanol medium, *i.e.* the representative C-doped ZnO sample. From the TEM images of the gel, which was treated at 200 °C for 3 h (Fig. 2a, b and S1†), it was confirmed that the gel consisted of a network of nanowires with 5–20 nm sized molecular assemblies. This gel network structure disintegrated at higher temperatures. At 300 °C, the mesh-like structure found at 200 °C had vanished (Fig. 2c, d and S2†). An amalgamate structure containing a carbonaceous unstructured layer and a growth unit of zinc oxide could be observed through the TEM image, and the approximate size of the seed nuclei was found to be 40 nm at 300 °C. The presence of spherical ZnO nanoclusters was evident from the TEM images at 400 °C/3 h (Fig. 2e, f and S3†), with an average size of 50 nm. As in the case of the samples at 500 °C (Fig. 2g, h and S4†), the average size of the particles was 60 nm. The representative SEM images of the carbon-doped ZnO nanoparticles at 400 °C/3 h further revealed that the bulk morphology of the prepared ZnO particles was that of spherical nanoclusters, with an average size of 50 nm (Fig. S5†).

### 3.3 Crystallographic analyses of C-ZnO, N-ZnO, and S-ZnO

**Fig. 3:**
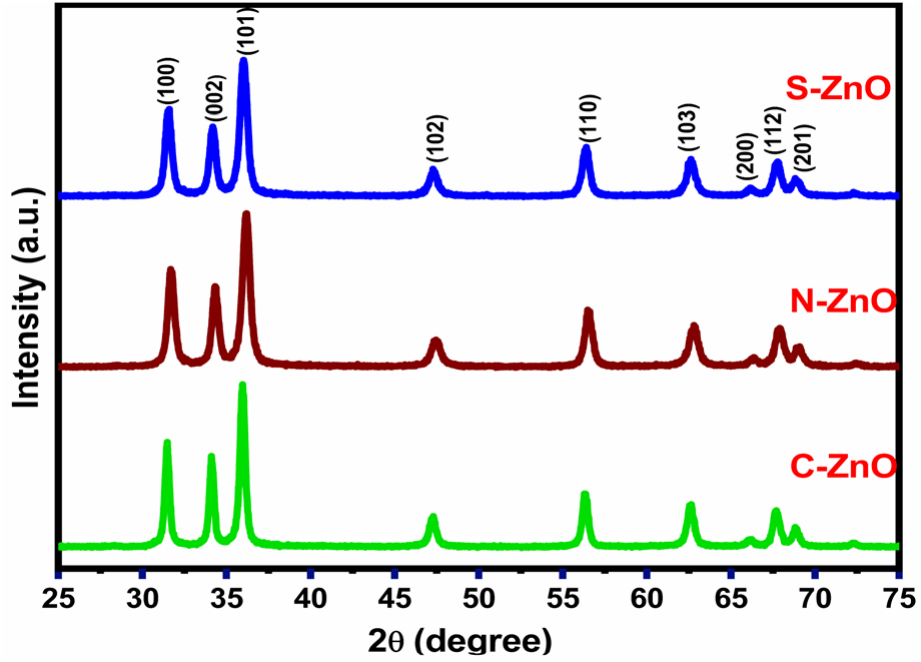
XRD patterns of C-, N-, and S-doped ZnO.

The X-ray diffraction pattern of the gel-derived ZnO samples from the C, N, and S precursors at 400 °C/3 h (Fig. S6†) are depicted in Fig. 3. As evidenced by the diffraction patterns, all the samples showed highly crystalline peaks of ZnO with a hexagonal wurtzite structure. The diffractogram of the prepared sample contained sharp and intense peaks at 31.78°, 34.23°, 36.95°, 46.86°, and 56.79°, which corresponded to the (100), (002), (101), (102), and (110) planes of ZnO.^38,40^ All the diffraction peaks confirmed the nanocrystalline nature of the prepared particles. No diffraction peaks of elements such as C, N, and S were especially detected, indicating that these elements substituted for Zn in the ZnO lattice.^41,42^ Upon the transition of the XRD pattern of C-ZnO from N-ZnO to S-ZnO, the ZnO samples' peaks in XRD (Fig. 1b) progressively shifted towards lower diffraction angles. The (100), (002), (101), (102), and (110) diffraction planes of N-ZnO were shifted to 31.72°, 34.37°, 36.22°, 47.49°, and 56.54°, respectively. Similarly, the (100), (002), (101), (102), and (110) diffraction planes of S-ZnO were shifted to 31.69°, 34.33°, 36.20°, 47.45°, and 56.50°, respectively. Introducing S ions into ZnO increased the values of the lattice constant and the lattice volume and shifted the diffraction peaks towards lower diffraction angles.^43,44^ The crystallite sizes of the obtained particles from the Debye–Scherer equation were 15, 12, and 10 nm for C-ZnO, N-ZnO, and S-ZnO, respectively.

### 3.4 Assessment of the chemical composition and the elemental analyses

**Fig. 4:**
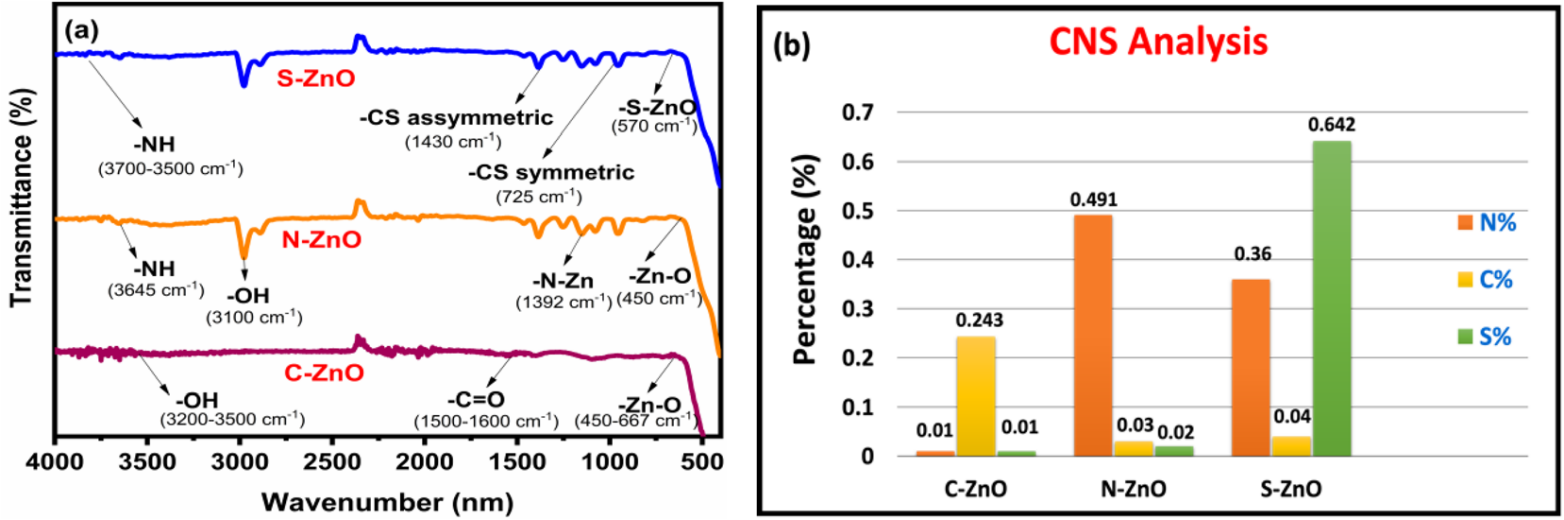
Structural analysis of C-ZnO, N-ZnO, and S-ZnO. (a) FT-IR spectra and (b) CNS analyses of the prepared samples.

Fig. 4a depicts the FT-IR spectroscopic analyses of the prepared samples in the wavelength range from 4000 to 400 cm^−1^. In the case of C-doped ZnO nanoparticles, a sharp band was observed at 3500–3200 cm^−1^, corresponding to the O-H stretching frequency, and the peak at 667–450 cm^−1^ indicated the Zn–O absorption band.^45^ A slight C=O stretching vibration was observed for this sample at 1500–1000 cm^−1^. The FT-IR spectra of N-doped ZnO NPs contained a peak at 3645 cm^−1^, corresponding to the N–H stretching frequency. The peak that appeared at 450 cm^−1^ corresponded to the Zn–O stretching vibration. The stretching vibration of O–H was pronounced at 3100 cm^−1^ due to the N-doping effect. The peak obtained at 1392 cm^−1^ provided evidence for the formation of the N–Zn bond. The FT-IR spectra of the S-doped ZnO nanoparticles exhibited the N–H absorption band in the region of 3700–3500 cm^−1^. The extended peaks at 725 and 1430 cm^−1^ corresponded to C-S symmetric and asymmetric stretching frequencies. The peak at 570 cm^−1^ corresponded to S-Zn stretching vibration. The FT-IR analyses finally affirmed the presence and bonding of C, N, and S in the prepared ZnO samples.

CNS analysis was used to determine the effectiveness of the present synthetic method for the *in situ* doping of carbon, nitrogen, and sulphur and the elemental composition of these elements in the prepared ZnO samples (Table 1). As provided in the bar diagram (Fig. 4b), the amount of C, N, and S compositions in the prepared samples varied according to the experimental conditions applied.

**Table 1:** Elemental composition of carbon, nitrogen, and sulphur in the prepared ZnO samples

Sample	C (%)	N (%)	S (%)
C-ZnO	0.243	0.010	0.010
N-ZnO	0.030	0.491	0.020
S-ZnO	0.040	0.360	0.642

### 3.5 XPS analysis of the non-metal-doped samples

The doping of elements such as N, S, and C, in the ZnO nanoparticles was further affirmed through the XPS spectra. Fig. 5.1, 5.2, and 5.3 present the survey spectra of the prepared samples and the high-resolution XPS spectrum of each element. The high-resolution XPS results are also provided in Fig. S7.† The survey spectrum of C-ZnO (Fig. 5.1) revealed the presence of three elements, namely Zn, O, and C, with their characteristic peaks corresponding to Zn 2p, C 1s, and O 1s.^46,47^ The XPS spectrum of Zn 2p showed two major peaks at 1018.80 and 1041.92 eV for the ZnO spin–orbit peaks of Zn 2p_3/2_ and Zn 2p_1/2_, respectively. The energy separation between Zn 2p_3/2_ and Zn 2p_1/2_ was thus 23.12 eV. The XPS spectra of N-ZnO exhibited two peaks at 1021.34 and 1044.53 eV for Zn 2p_3/2_ and Zn 2p_1/2_, respectively. The XPS spectrum of Zn 2p for S-ZnO showed two strong peaks at 1021.28 and 1045.26 eV for Zn 2p_3/2_ and Zn 2p_1/2_, respectively. Upon comparing the XPS spectrum of Zn 2p to C-ZnO and N-ZnO, the binding energy of S-ZnO was found to be increased. The binding energy differences were measured to be 23.12, 23.53, and 24.00 eV in the cases of C-ZnO, N-ZnO, and S-ZnO, respectively.

**Fig. 5:**
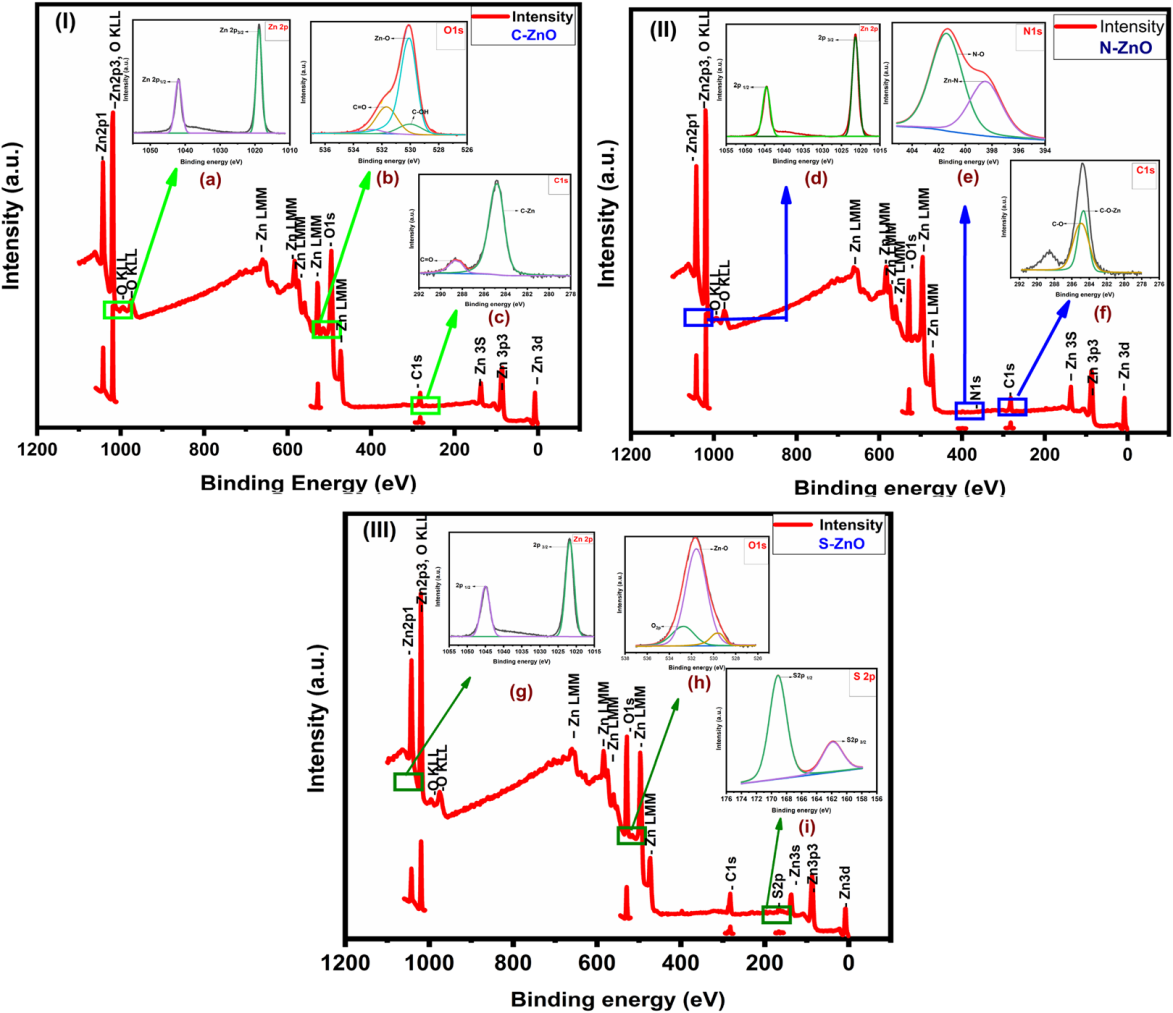
XPS analyses of the prepared samples; XPS survey spectra of: (5.1) C-ZnO, (5.2) N-ZnO, and (5.3) S-ZnO. Insets are the high-resolution XPS spectra of: (a) Zn 2p, (b) O 1s & (c) C 1s of C-ZnO. (d) Zn 2p, (e) N 1s, and (f) C 1s of N-ZnO, (g) Zn 2p, (h) O 1s, and (i) S 2p of S-ZnO.

Fig. 5.1b further illustrates the XPS spectra for O 1s. The bonds corresponding to Zn–O, C–OH, and C=O were defined with their characteristic peak at 530.00, 529.83, and 532.00 eV, respectively. The hexagonal wurtzite structure of ZnO was proven by the bond between ZnO, which appeared as a highly intense peak at 530.00 eV. The other peaks observed at 529.83 and 532.00 eV were contributed by the oxygen atom in the crystal shared with the carbon atom, which could be understood from the formation of C–OH and C=O bonds, respectively. The O 1s spectrum of S-ZnO (Fig. 5.2h) was deconvoluted into two major peaks corresponding to Zn–O and O 2p. The peak at 531.00 eV, corresponding to the Zn–O bond, indicated that S-ZnO had a hexagonal wurtzite structure. The peak at 532.72 eV was the characteristic peak of O 2p, which indicated that O^2−^ ions were adsorbed on the ZnO crystal structure along with the Zn^2+^ ion. Fig. 5.2e shows the N 1s spectrum of N-ZnO. From the N 1s spectrum, the proper doping of N in ZnO was proven from the peak at 398.54 eV due to the Zn–N bond and at 401.46 eV corresponding to the N–O bond.

Fig. 5.1c and 5.2f correspond to the high-resolution XPS spectra of C 1s in the case of C-ZnO and N-ZnO, respectively. The C 1s spectra were deconvoluted into two major peaks at 285.50 and 284.80 eV, corresponding to the C=O and C–Zn bonds, respectively. As in the case of the C 1s spectrum for the sample N-ZnO, the peaks centered at 284.62 and 288.55 eV corresponded to C–O–Zn and C–O bonds, respectively. Fig. 5.2i presents the S 2p spectrum of S-ZnO, which could be resolved into two significant peaks at 169.00 and 161.83 eV corresponding to S 2p_1/2_ and S 2p_3/2_ respectively, indicating the sulphur atom was bound to the ZnO crystal surface in the form of S^2−^. The XPS data thus further affirmed the presence of structurally bonded C, N, and S in the prepared ZnO nanoparticles.

### 3.6 Porosity and surface area analyses

**Fig. 6:**
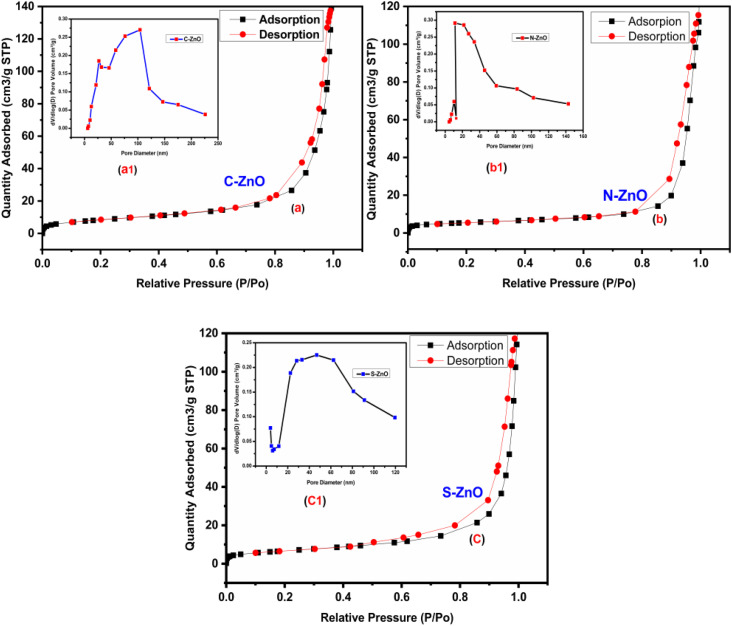
BET surface area plots of (a) C-ZnO, (b) N-ZnO, and (c) S-ZnO catalysts with N_2_ adsorption–desorption isotherms; insets show the pore-size distribution plots for (a1) C-ZnO, (b1) N-ZnO, and (c1) S-ZnO.

The pore-size distributions and surface area of the prepared C-ZnO, N-ZnO, and S-ZnO samples were studied through the BET sorption method using N_2_ gas, as illustrated in Fig. 6. From the BET method, the surface areas of C-ZnO, N-ZnO, and S-ZnO were calculated to be 31, 19, and 24 m^2^ g^−1^, respectively. The pore-size distributions of C-ZnO (35 nm), N-ZnO (54 nm), and S-ZnO (67 nm), shown in the insets in Fig. 6a–c, clearly illustrate that S-ZnO and N-ZnO had macroporous structures, while C-ZnO had a mesoporous nature.^48–50^ The porosity profile of ZnO nanoparticles extends their application possibility as a good photocatalyst in photocatalytic applications.

### 3.7 Diffused reflectance spectra

**Fig. 7:**
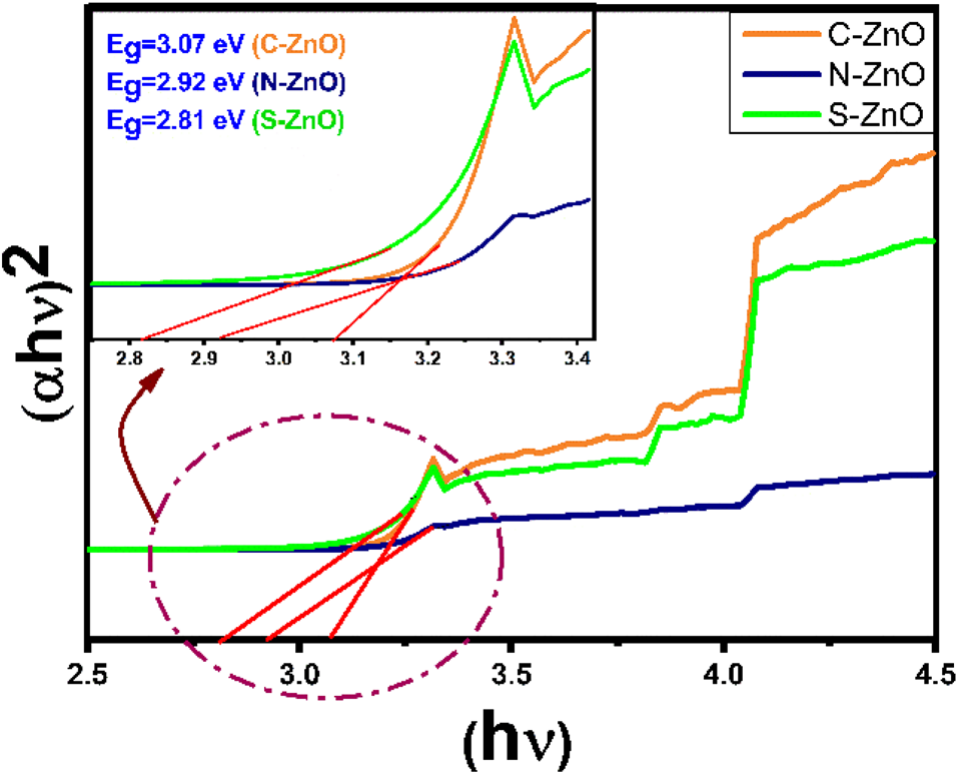
UV–vis Kubelka–Munk diffuse reflectance spectra (Tauc plots) of C-doped ZnO, N-doped ZnO, and S-doped ZnO.

UV–Vis diffuse reflectance spectrophotometric analyses were carried out for the present samples, and Kubelka–Munk plots were constructed, which are depicted in Fig. 7. The Kubelka–Munk mathematical function was applied to determine the energy gap, *E*_g_, of the samples. According to the Kubelka–Munk function, the relationship between the absorption coefficient (*K*), scattering or dispersion coefficient (*S*), and the fraction of the reflected light (*R*) can be expressed as,

1 *F*(*R*) = *K*/*S* = (1 − *R*)^2^/2*R*

from the plots, the band gap energies of C-ZnO, N-ZnO, and S-ZnO were calculated as 3.07, 2.92, and 2.81 eV, respectively. The band gaps obtained for the present cases were much less than the actual band gap of ZnO of 3.37 eV.^51^ The reason behind the lower band gap energy of C-, N-, and S-doped ZnO was likely due to the structural defects and the alternations that happened in the lattice due to the non-metal inclusions. The presence of N and S was expected to provide sub-bands above the valence band of ZnO, which may ultimately be caused by the decreased band gap energy.^52^ The S-ZnO sample had the lowest ‘*E*_g_’ value when compared with the other two samples, indicating its high photoabsorption efficiency, particularly in the visible region.

### 3.8 Photocatalytic activity of the C-, N-, and S-doped ZnO

**Fig. 8:**
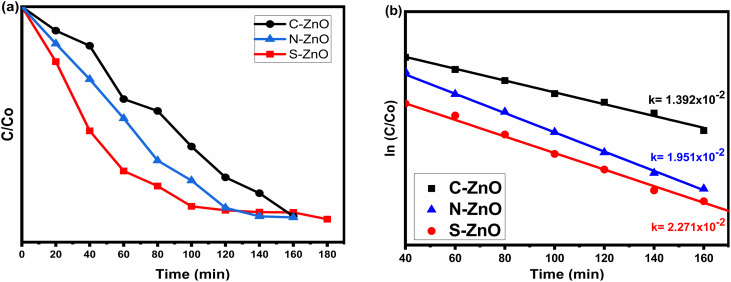
Photocatalytic performances of C-, N-, and S-doped ZnO under UV light. (a) *C*/*C*_0_*vs.* time plot, (b) ln *C*/*C*_0_*vs.* time plot showing the photocatalytic degradation constants.

Methylene blue (MB) dye was used as the organic pollutant to study the photocatalytic degradation efficiency and the environmental remediation capability of the prepared samples. The degradation efficiency was tested for C-doped ZnO, N-doped ZnO, and S-doped ZnO photocatalysts. The MB dye samples were taken from the photoreactor (Fig. S8†) at 20 min intervals of light exposure, and the UV–visible spectrum was recorded (Fig. S9†). The absorption maximum of MB dye was reported at 667 nm. The kinetics of degradation of MB is depicted through the graph of *C*/*C*_0_ and ln(*C*/*C*_0_) *versus* time (Fig. 8a and b), where *C*_0_ is the initial concentration of MB and *C* is the concentration of MB after a particular time.

The photocatalytic degradation efficiencies of the prepared samples were evaluated using the following equation,^53^2
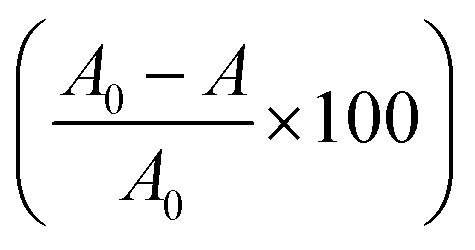
where *A*_0_ is the absorbance of the solution of the dye before the irradiation of photons, and *A* is the absorbance of the dye solution after the irradiation of photons at a particular time. The photocatalytic degradation efficiencies of C-ZnO, N-ZnO, and S-ZnO were found to be 67.2%, 78.5%, and 81.4%, respectively, with 3 h of irradiation. The photocatalytic degradation of MB followed a pseudo-first-order kinetics, and the values of the correlation coefficients (*R*^2^) were obtained as nearly 1. The photocatalytic degradation constants (*k* or *k* apparent) obtained for S-ZnO, N-ZnO, and C-ZnO were 2.271 × 10^−2^, 1.951 × 10^−2^, and 1.392 × 10^−2^ min^−1^ respectively. All the samples obtained through the present method showed higher photodegradation efficiency when compared to the standard undoped ZnO (Table S1†). Thus, by investigating the photodegradation of MB in the presence of the C-ZnO, N-ZnO, and S-ZnO photocatalysts under UV–visible light illumination, it was revealed that S-ZnO had a more remarkable ability to degrade MB to a greater extent than N-ZnO, which in turn showed better activity than C-ZnO, for the samples prepared through the same gelation and thermal treatment methods. Notably, solar-light-active photocatalysts with varying catalytic efficiency were finally obtained. This may further explain the robustness of the present *in situ* approaches too for synthesizing doped ZnO materials for photocatalytic/environmental remediation applications.

Environmental contamination is often remediated by non-metal-doped ZnO.^54,55^ Numerous studies have noted that doping ZnO with S is one of the best ways to alter its structural and optical features because of the substantial electronegativity and size difference between S and O.^56–59^ It was discovered that the structural, morphological, luminescent, and photocatalytic characteristics of ZnO are also influenced by the molar ratio of Zn to S. The difference in the electrical and optical characteristics of semiconductor photocatalysts can be attributed to variations in the lattice constant, which is influenced by the replacement of S for O since S^2−^ has a bigger size than O^2−^.

Additionally, the concentrations of S and O vacancies, as revealed through XPS analyses of the samples, affect the photocatalytic activity of S-doped ZnO.^56,59^ During the photocatalytic process, O vacancies turn into centers for capturing photoinduced electrons, which is important since an increase in oxygen vacancies can lead to an increase in photocatalytic activity. Additionally, oxygen vacancies facilitate the adsorption of O_2_ and produce superoxide radicals when they interact with photoinduced electrons.^59^ The S-ZnO nanoparticles effectively act as electronic traps and prevent the recombination of charge carriers. Also, they can transport electrons onto the oxygen molecules to produce superoxide radicals (˙O_2_^−^) over the catalytic active sites. On the other hand, photoinduced active intermediate radicals, like ˙O_2_^−^ and OH˙, directly take the electrons of MB existing over the photocatalyst surface and convert the dye molecules to various degradation compounds (eqn (3)–(9)).^17,19,20,22,33,60,61^

3 S-ZnO + *hν* → S-ZnO (e^−^ + h^+^)

4 S-ZnO(h^+^) + H_2_O → ˙OH + H^+^

5 O_2_ + S-ZnO(e^−^) → ˙O_2_^−^

6 dye + *hν* → dye*

7 dye* + S-ZnO(h^+^) → S-ZnO + dye^+^

8 dye (or dye^+^) + ˙O_2_^−^ → degraded compounds

9 dye (or dye^+^) + OH˙ → degraded compounds

Defects and vacancies are also caused by incorporating S into ZnO.^55,60^ It was noted that the O vacancies increased when the S content rose. Under solar radiation, S-ZnO has a higher electrical conductivity due to the increase in O vacancies.^60,62^ Due to the increased transport of photoinduced charge carriers and increased light-absorption capacity of photocatalysts in the visible-light region, S doping is an effective method for reducing the band gaps of semiconductor oxides and assisting in the shifting of the threshold wavelength towards the UV/visible-light region.^63,64^ This promotes an increase in photocatalytic activity. Since carbon (C) and nitrogen (N) have atomic radii that are comparable to those of oxygen (O), adding these non-metals causes the band gap of ZnO to narrow slightly, although not as much as for S-ZnO. Also, S-ZnO exhibited significant electronegativity and size differences between S and O, when compared with C-ZnO and N-ZnO, which may also contribute to its comparative photocatalytic performance.^60,64^

### 3.9 Comparative photocatalytic performance diagram and the mechanisms involved

**Fig. 9:**
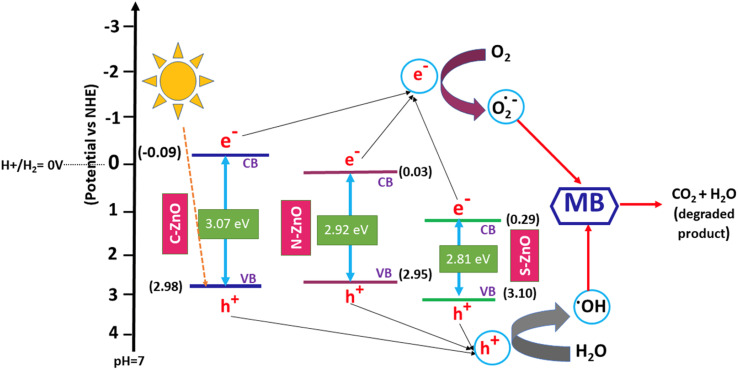
Comparative photocatalytic performances diagram of C-, N-, and S-doped ZnO for methylene blue dye degradation.

From the photocatalytic performance studies, the influence of the non-metal dopants in the band gap engineering of ZnO was revealed. In the present case, unlike the pure valence band to conduction band electron promotion happening in pure ZnO, the electron transition could take place from many sub-bands of ZnO. As shown in the comparative photocatalytic performance diagram in Fig. 9, electron transitions could occur from different ZnO sub-bands, for the C-, N-, and S-doped ZnO. This explains the lower band gap values of the doped system, as evidenced by the diffused reflectance spectra. From the XPS spectra, the valence band maxima of C-ZnO, N-ZnO, and S-ZnO were 2.98, 2.95, and 3.10 eV, respectively (Fig. S10†). Upon comparing the values of the band gap energy calculated using the UV–Vis. diffuse reflectance spectrophotometric analyses, the values of the conduction band minima of C-ZnO, N-ZnO, and S-ZnO were calculated to be −0.09, 0.03, and 0.29 eV, respectively. Thus, the effective band gap displayed by the samples C-ZnO, N-ZnO, and S-ZnO were 3.07, 2.92, and 2.81 eV, respectively.

As illustrated in Table 2, in comparison with C- and N-doped ZnO, the S-doped ZnO had a small crystallite size (10 nm), low band gap energy (2.81 eV), and a big pore-size distribution (67 nm). These factors ultimately contributed to the high photocatalytic activity of the sample, which resulted in a good degradation efficiency of 81.4% and a high degradation constant of 2.271 × 10^−2^. In the case of N-doped ZnO, the sample exhibited a low crystallite size (12 nm), low band gap energy (2.92 eV), and medium pore size (54 nm). These values followed its photocatalytic performances, which revealed a better degradation efficiency of 78.5% and a high degradation constant of 1.951 × 10^−2^. However, when compared with the S- and N-doped ZnO systems, carbon-doped ZnO displayed a low dye degradation efficiency of 67.2% and a degradation constant of 1.392 × 10^−2^.

**Table 2:** Comparative crystallite sizes, band gap energies, pore-size distributions, degradation efficiencies, and degradation constants of the C-, N-, and S-doped ZnO photocatalysts

Photocatalyst	Crystallite size (nm)	Band gap energy (eV)	BET pore-size distribution (nm)	MB dye degradation efficiency (%)	Degradation constant, *k* (min^−1^)
C-ZnO	15	3.07	35	67.2	1.392 × 10^−2^
N-ZnO	12	2.92	54	78.5	1.951 × 10^−2^
S-ZnO	10	2.81	67	81.4	2.271 × 10^−2^

The present study thus clearly illustrates that graded ZnO photocatalysts with varying catalytic efficiencies can be prepared by the selective doping of non-metals through conventional heating. The present method was found to be an industrially sustainable chemical approach since it eventually produced doped ZnO nanoparticles sized less than 50 nm at a low temperature of 400 °C/3 h. Bulk scales of doped nanoparticles could be prepared by adopting this simple processing technique. Solar photocatalysts prepared through the present method exhibited a spherical nanocluster morphology, lower crystallite size, low band gap energy, high surface area, and good porosity features. The synergy between the preparation method, the nature of dopants, and the band gap engineering expands the possibility of the present preparation method for developing non-metal-doped ZnO solar photocatalysts, which are highly beneficial for many sustainable chemical and green industrial applications, like water purification and environmental remediation.

## 4 Conclusions

Sustainable, non-metal (C, N, and S)-doped nanocrystalline ZnO photocatalysts with high catalytic efficiency were prepared through a simple thermo-evolution method. The characterization studies using XRD and TG/DTA analyses confirmed the thermo-evolution of the nanocrystalline ZnO at 400 °C. The morphological studies performed using TEM and SEM analyses gave full proof evidence of the structural changes and conversion of the morphologies, from mesh-like nanowires (200 °C) to nanodots (300 °C) to spherical clusters (400 °C) of ZnO. The thermally derived nanoparticles exhibited a spherical morphology and an average particle size of <50 nm, at 400 °C/3 h. Irrespective of the type of non-metal dopants present, the diffractogram of the prepared non-metal doped ZnO samples exhibited only sharp and intense peaks corresponding to the (100), (002), (101), (102), and (110) planes of ZnO. The Debye–Scherer crystallite sizes of the obtained particles were measured to be 15, 12, and 10 nm for C-ZnO, N-ZnO, and S-ZnO, respectively. The FT-IR fingerprint bands, corresponding to N-Zn at 1392 cm^−1^, C-S symmetric and asymmetric stretching at 725 and 1430 cm^−1^, and the S-Zn stretching band at 570 cm^−1^, evidenced the presence of structurally bonded non-metals in the present samples. The CNS analyses further quantified the presence of carbon, nitrogen, and sulphur in the prepared samples. The binding energy associated with and the type of structurally bonded C-, N-, and S-doped ZnO were evaluated by XPS analyses. From the UV-Vis-diffuse reflectance Kubelka–Munk plots, the band gap energies of C- ZnO, N-ZnO, and S-ZnO were calculated as 3.07, 2.92, and 2.81 eV, respectively. The lower band gap values of these samples pinpoint the enhanced light-absorption capability of these samples in the UV–visible region. The synergy between the merits of the preparation method and the band gap engineering was revealed in the photocatalytic efficiencies of the samples. The environmental remediation application studies showed that the photocatalytic dye degradation efficiencies of C-ZnO, N-ZnO, and S-ZnO were 67.2%, 78.5%, and 81.4% respectively, with the photocatalytic degradation constants (*k*) of these samples as 1.392 × 10^−2^, 1.951 × 10^−2^, and 2.271 × 10^−2^ min^−1^. The comparative photocatalytic performances confirmed that the S- and N-doped ZnO photocatalysts had better photoactivity than the C-doped ZnO, due to their lower crystallite size, lower band gap energy, and big pore-size distribution. The possible mechanistic pathway was illustrated using XPS and UV-Vis-diffuse reflectance, which suggested that electron transitions could occur from different ZnO sub-bands if C-, N-, and S-based non-metals were doped to ZnO. The study affirms that, even for the C-doped ZnO, nanoparticles were obtained with a lower crystallite size value (15 nm), mesoporous nature, and a low band gap energy (3.07 eV) value, which explains the efficacy of the present preparation method when compared with other preparation methods. Highly efficient solar photocatalysts prepared through the present methods should thus find many futuristic green industrial applications.

## Data availability

Data will be made available on request.

## Author contributions

Amala Joy: writing – original draft, methodology, investigation. Mangalaraja R. Viswanathan: validation, formal analysis, data curation. Baiju K. Vijayan: resources, investigation, formal analysis. Claudia G. Silva: writing – review & editing, investigation. Irfana Basheer: conceptualization, formal analysis. Sreejamol Sugathan: methodology, investigation. Peer A. Muhammed: resources, formal analysis. Ananthakumar Solaippan: writing – review & editing, supervision, resources. Anas Shereef: supervision, writing – review & editing, resources, fund acquisition.

## Conflicts of interest

There are no conflicts of interest to declare.

## Supplementary Material

RA-014-D4RA03492A-s001
