# Supplementary material for: Solar photocatalysts: non-metal (C, N, and S)-doped ZnO synthesized through an industrially sustainable in situ approach for environmental remediation applications
Source: RSC Adv. 2024 Jul 8;14(30):21655–67. doi: 10.1039/d4ra03492a (PMC11229408; doi:10.1039/d4ra03492a)
Supplement: RA-014-D4RA03492A-s001 [file RA-014-D4RA03492A-s001.pdf]

Electronic Supplementary Material (ESI) for RSC Advances.
This journal is © The Royal Society of Chemistry 2024

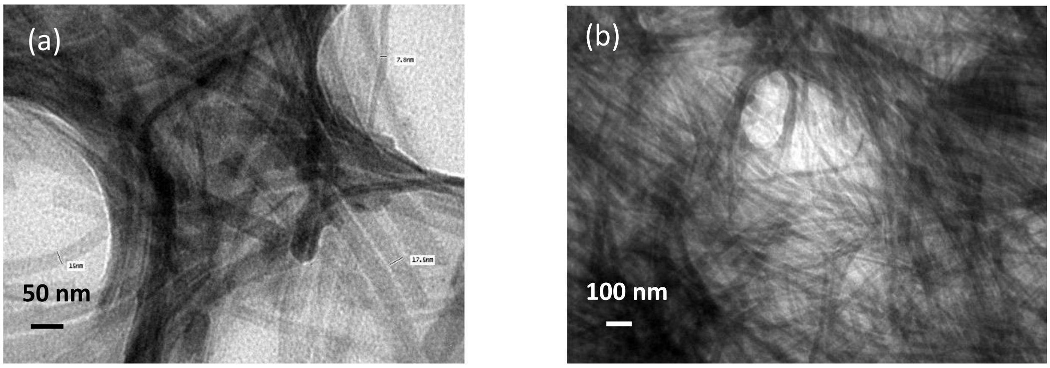

Fig. S1 TEM images of zinc acetate-tartaric acid (1:1) gels (a) & (b) [200 °C/3h].

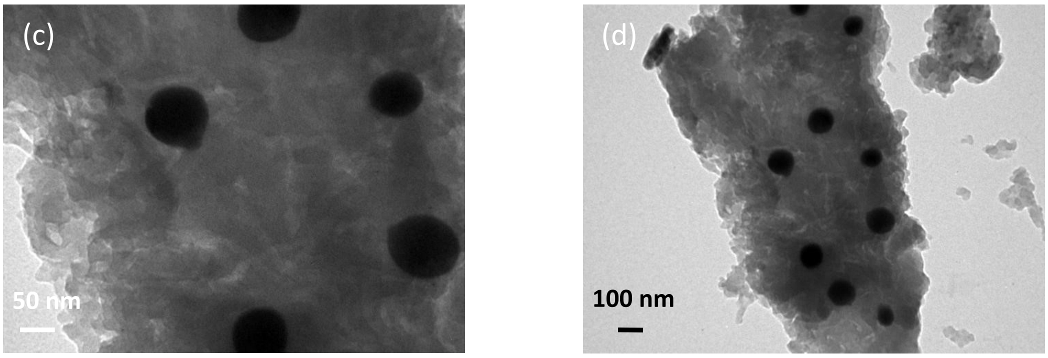

Fig. S2 TEM images of zinc acetate-tartaric acid (1:1) gels (c) & (d) [300 °C/3h].

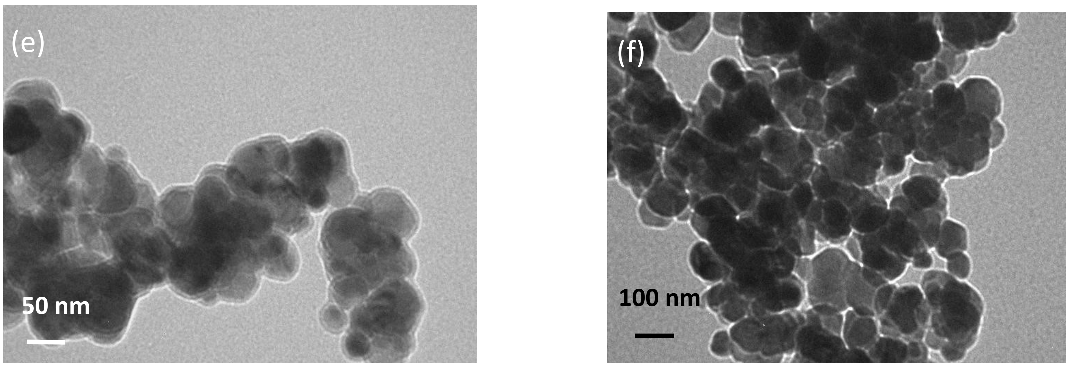

Fig. S3 TEM images of zinc acetate-tartaric acid (1:1) gels (e) & (f) [400 °C/3h].

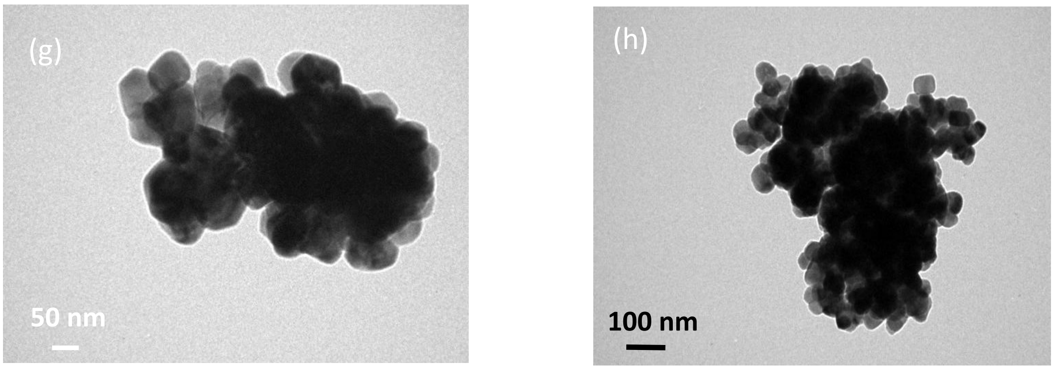

Fig. S4 TEM images of zinc acetate-tartaric acid (1:1) gels (g) & (h) [500 °C/3h].

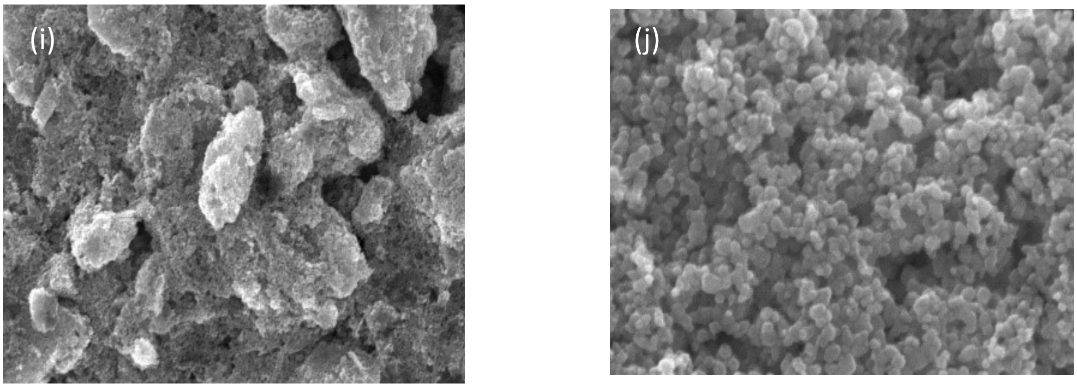

Fig. S5 SEM images of C-doped zinc oxide nanoparticles at low (a) and high (b) magnifications.

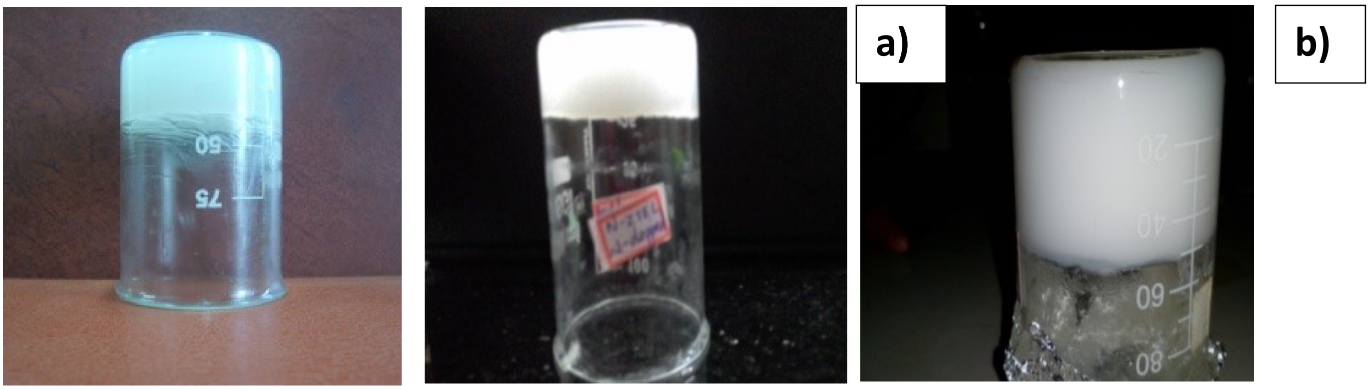

Fig. S6 Photographic images a) C-ZnO b) N-ZnO & c) S-ZnO gel sample.

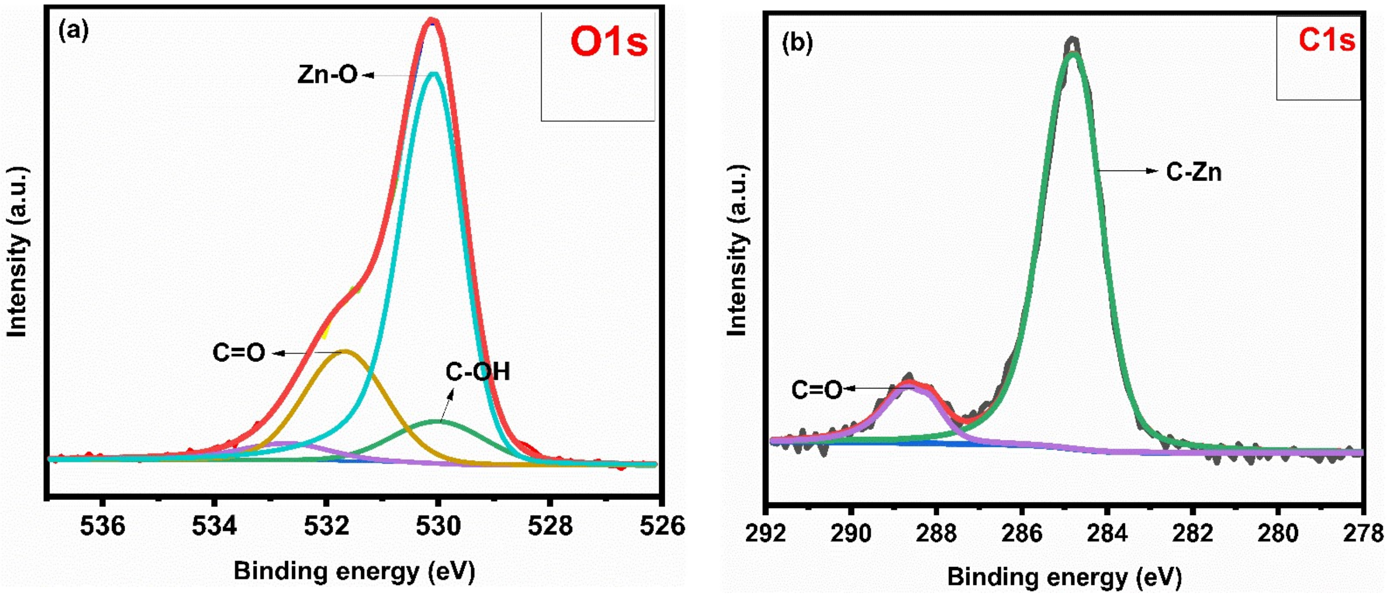

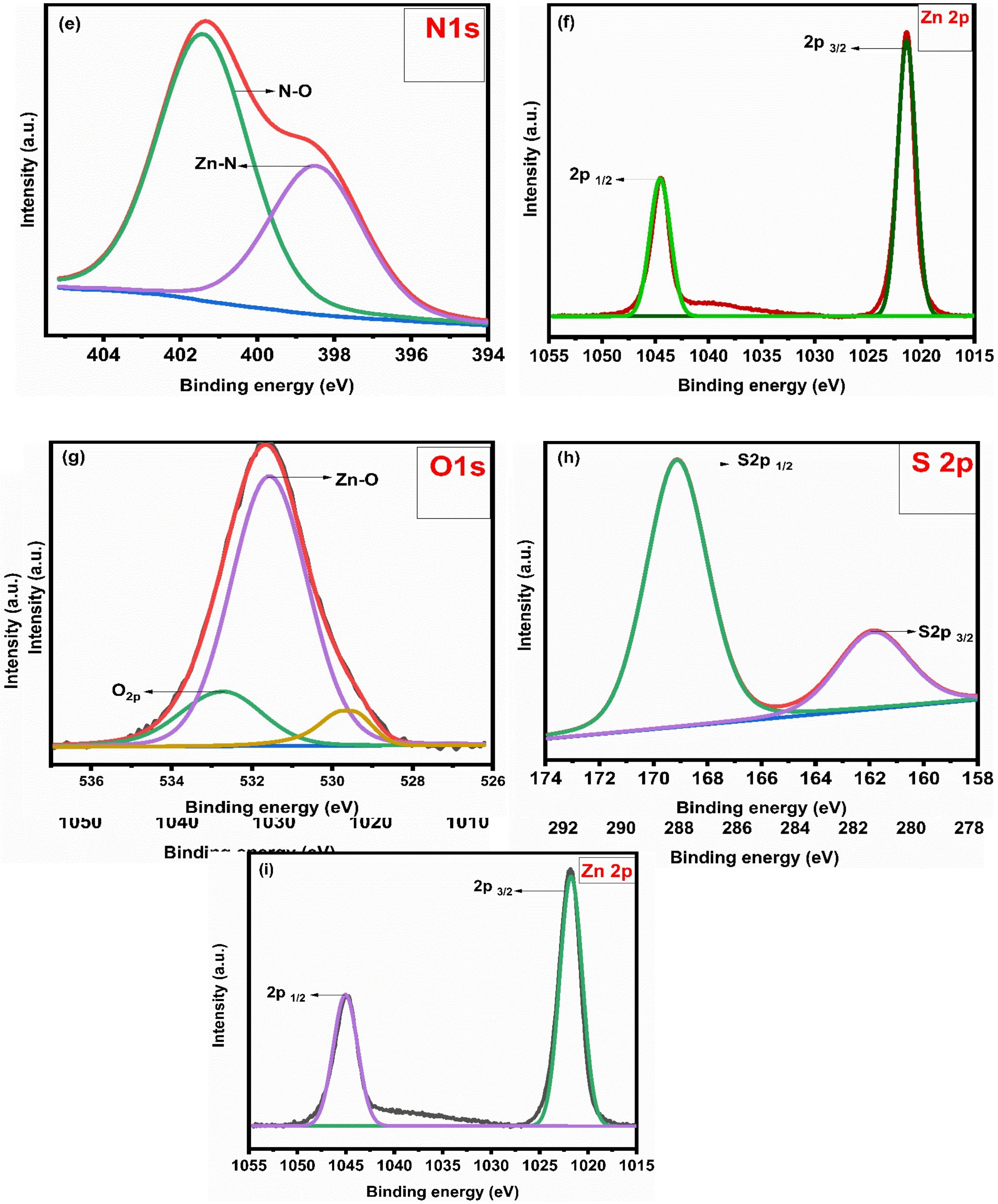
(e)
N1s
N-O
Zn-N
Intensity (a.u.)
404 402 400 398 396 394
Binding energy (eV)
(f)
Zn 2p
2p 3/2
2p 1/2
Intensity (a.u.)
1055 1050 1045 1040 1035 1030 1025 1020 1015
Binding energy (eV)
(g)
O1s
Zn-O
O2p
Intensity (a.u.)
536 534 532 530 528 526
Binding energy (eV)
1050 1040 1030 1020 1010
(h)
S 2p
S2p 1/2
S2p 3/2
Intensity (a.u.)
174 172 170 168 166 164 162 160 158
Binding energy (eV)
292 290 288 286 284 282 280 278
Binding energy (eV)
(i)
Zn 2p
2p 3/2
2p 1/2
Intensity (a.u.)
1055 1050 1045 1040 1035 1030 1025 1020 1015
Binding energy (eV)

Fig. S7 High-resolution XPS spectra of (a) O1s, (b) C1s & (c) Zn 2p of C-ZnO. (d) C1s, (e) N1s and (f) Zn 2p of N-ZnO, (g) O1s, (h) C1s and (i) Zn 2p of S-ZnO.

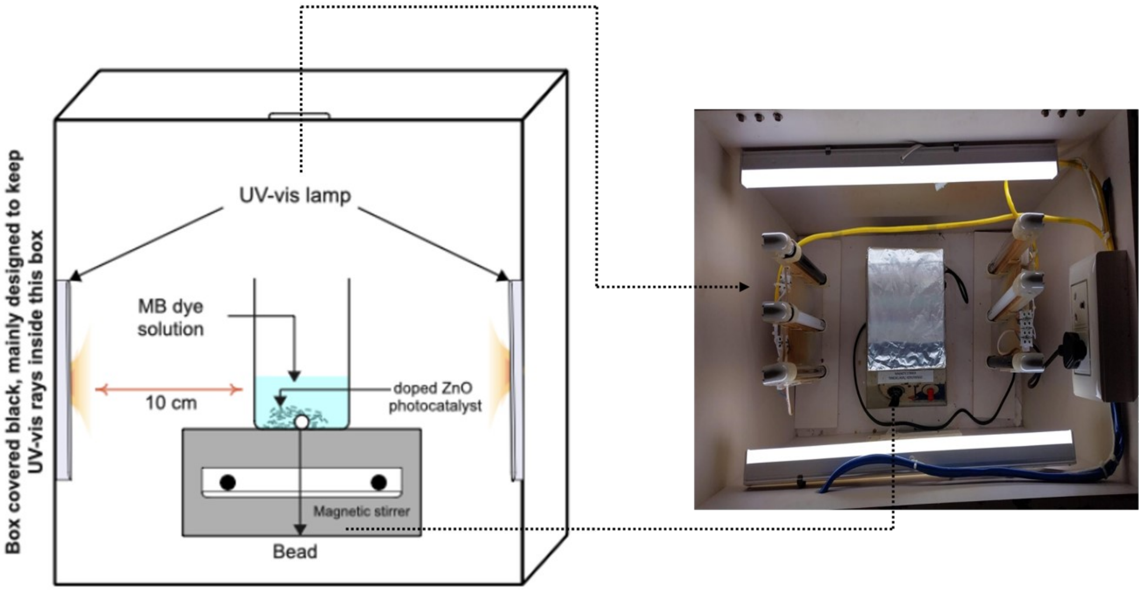

Fig. S8 Image of the photoreactor used for the photocatalytic degradation of MB.

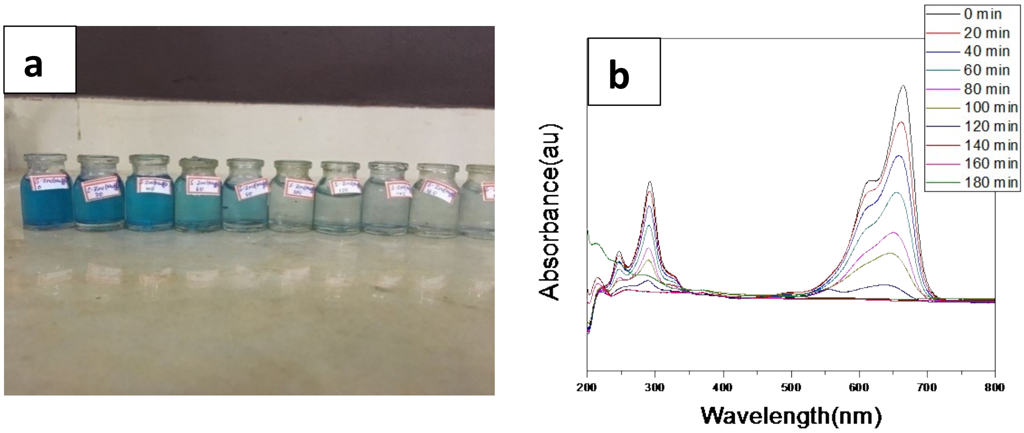

Fig. S9 Representative images of the photocatalytic degradation of methylene blue (a) and the degradation profile taken using UV-vis. spectrophotometer (b) for the S-doped ZnO samples.

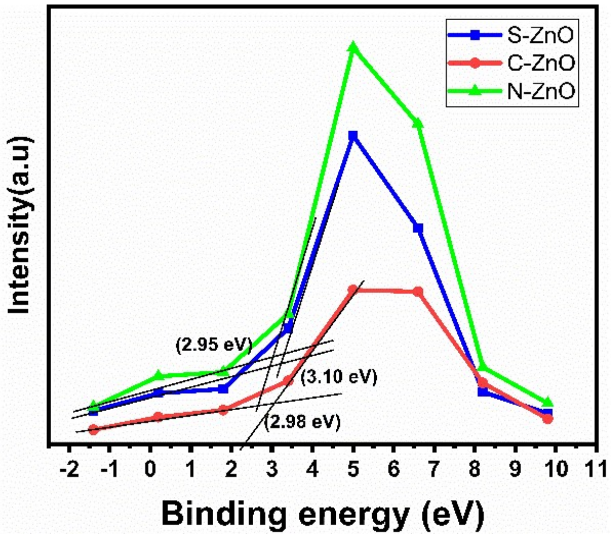

Fig. S10 Valence Band (VB) energy levels from XPS spectra.

Table S1 Apparent rate constant (k) and R^2 value for degradation of MB in the presence of doped (C, N and S) and undoped ZnO Nanoparticles.

Photocatalysts	k x 10^{-2} (min^{-1}) ($k_{apparent}$)	R^2 (correlation coefficients)
C-ZnO	1.392	0.99566
N-ZnO	1.951	0.99761
S-ZnO	2.271	0.99902
ZnO (Standard)	0.830	0.92060
